# Composition of the adult digestive tract bacterial microbiome based on seven mouth surfaces, tonsils, throat and stool samples

**DOI:** 10.1186/gb-2012-13-6-r42

**Published:** 2012-06-14

**Authors:** Nicola Segata, Susan Kinder Haake, Peter Mannon, Katherine P Lemon, Levi Waldron, Dirk Gevers, Curtis Huttenhower, Jacques Izard

**Affiliations:** 1 Department of Biostatistics, 677 Huntington Avenue, Harvard School of Public Health, Boston, MA 02115, USA; 2 Section of Periodontics, UCLA School of Dentistry, 10833 Le Conte Ave, Los Angeles, CA 90095, USA; 3 Dental Research Institute, UCLA School of Dentistry, 10833 Le Conte Ave, Los Angeles, CA 90095, USA; 4 Division of Gastroenterology and Hepatology, University of Alabama at Birmingham, 1825 University Boulevard, Birmingham, AL 35205, USA; 5 Department of Molecular Genetics, 245 First Street, The Forsyth Institute, Cambridge, MA 02142, USA; 6 Division of Infectious Diseases, Children's Hospital Boston, Harvard Medical School, 300 Longwood Avenue, Boston, MA 02115, USA; 7 Microbial Systems and Communities, Genome Sequencing and Analysis Program, The Broad Institute of MIT and Harvard, 7 Cambridge Center, Cambridge, MA 02142, USA; 8 Department of Oral Medicine, Infection and Immunity, 188 Longwood Ave, Harvard School of Dental Medicine, Boston, MA 02115, USA

## Abstract

**Background:**

To understand the relationship between our bacterial microbiome and health, it is essential to define the microbiome in the absence of disease. The digestive tract includes diverse habitats and hosts the human body's greatest bacterial density. We describe the bacterial community composition of ten digestive tract sites from more than 200 normal adults enrolled in the Human Microbiome Project, and metagenomically determined metabolic potentials of four representative sites.

**Results:**

The microbiota of these diverse habitats formed four groups based on similar community compositions: buccal mucosa, keratinized gingiva, hard palate; saliva, tongue, tonsils, throat; sub- and supra-gingival plaques; and stool. Phyla initially identified from environmental samples were detected throughout this population, primarily TM7, SR1, and Synergistetes. Genera with pathogenic members were well-represented among this disease-free cohort. Tooth-associated communities were distinct, but not entirely dissimilar, from other oral surfaces. The Porphyromonadaceae, Veillonellaceae and Lachnospiraceae families were common to all sites, but the distributions of their genera varied significantly. Most metabolic processes were distributed widely throughout the digestive tract microbiota, with variations in metagenomic abundance between body habitats. These included shifts in sugar transporter types between the supragingival plaque, other oral surfaces, and stool; hydrogen and hydrogen sulfide production were also differentially distributed.

**Conclusions:**

The microbiomes of ten digestive tract sites separated into four types based on composition. A core set of metabolic pathways was present across these diverse digestive tract habitats. These data provide a critical baseline for future studies investigating local and systemic diseases affecting human health.

## Background

The bacterial microbiome of the human digestive tract contributes to both health and disease. In health, bacteria are key components in the development of mucosal barrier function and in innate and adaptive immune responses, and they also work to suppress establishment of pathogens [1]. In disease, with breach of the mucosal barrier, commensal bacteria can become a chronic inflammatory stimulus to adjacent tissues [2,3] as well as a source of immune perturbation in conditions such as atherosclerosis, type 2 diabetes, non-alcoholic fatty liver disease, obesity and inflammatory bowel disease [4-8]. It is therefore critically important to define the microbiome of healthy persons in order to detect significant variations both in disease states and in pre-clinical conditions to understand disease onset and progression.

The Human Microbiome Project (HMP) established by the National Institutes of Health aims to characterize the microbiome of a large cohort of normal adult subjects [9], providing an unprecedented survey of the microbiome. The HMP includes over 200 subjects and has collected microbiome samples from 15 to 18 body habitats per person [10]. This unique dataset permits novel studies of the human digestive tract within a large number of subjects, allows for comparisons of microbial communities between habitats, and enables the definition of distinct metabolic niches within and among individuals. Previous studies of the healthy adult digestive tract microbiota have typically included less than 20 individuals [11-21] and the studies with over 100 individuals have most often focused on a single body site [22-26]. The increased throughput, the improved sensitivity of assays and the improvements in next generation sequencing technologies have enabled cataloging of microbial community membership and structure [12,19,27] as well as the metagenomic gene pool present in each community in large numbers of samples from large numbers of subjects. The HMP in particular includes, for each sample, both 16S rRNA gene surveys and shotgun metagenomic sequences, from a subset of the subjects recruited at two geographically distant locations in the United States. The recruitment criteria included a set of objective, composite measurements performed by healthcare professionals [10], defining this reference population and enabling this investigation to focus on defining the integrated oral, oropharyngeal, and gut microbiomes in the absence of host disease.

The focus of this study, complementary to other activities in the HMP consortium, was to measure and compare the composition, relative abundance, phylogenetic and metabolic potential of the bacterial populations inhabiting multiple sites along the digestive tract in the defined adult reference HMP subject population. The digestive tract was represented by ten microbiome samples from distinct body habitats: seven samples were from the mouth (buccal mucosa, keratinized attached gingiva, hard palate, saliva, tongue and two surfaces along the tooth); two oropharyngeal sites (back wall of the oropharynx (refered to here as throat) and the palatine tonsils); and the colon (stool). In addition to their distinct anatomic locations, these sites were chosen because sampling minimally disturbed the existing micobiota and involved minimal risk to participants. Although existing data indicate that mucosa-associated communities below the pharynx may have distinct microbiomes, these sites were not included, as sampling would have required invasive procedures [16,17,28].

The results show that the ten body habitats examined here formed four categories of microbial community types. These four community types included taxa typically classified as 'environmental' phyla. Genera characterized by pathogenic species and thus associated with disease were also widely distributed among the population. Most striking, each body site (within as well as between the four groups) possessed a highly distinctive community structure with moderate variability across the population, and with distinct abundances of some microbial metabolic processes within each community. The combination of high-throughput sequencing technologies and a large, well-characterized study population has thus provided quantitative and qualitative outputs that allow a comprehensive definition of the normal adult digestive tract microbiome.

## Results

### Microbial community structure indicates four distinct community types within the ten digestive tract sites

**Figure 1:**
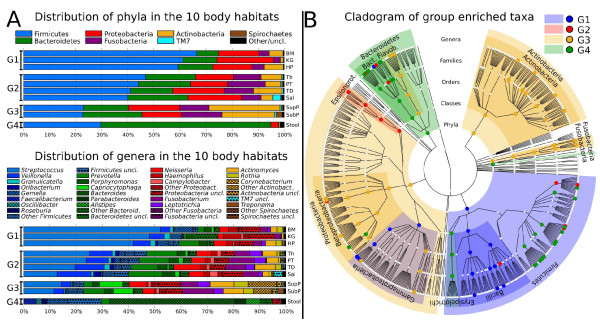
**Groups detected in the sampled digestive tract microbiome sites based on similarities in microbial composition**. **(a) **Taxonomic composition of the microbiota in the ten digestive tract body habitats investigated based on average relative abundance of 16S rRNA pyrosequencing reads assigned to phylum (upper chart) and genus (lower chart). Microbiota from the ten habitats are grouped based on the ratio of Firmicutes to Bacteroidetes as follows: Group 1 (G1), buccal mucosa (BM), keratinized gingiva (KG) and hard palate (HP); Group 2 (G2), throat (Th), palatine tonsils (PT), tongue dorsum (TD) and saliva (Sal); Group 3 (G3), supraginval (SupP) and subgingival plaques (SubP); and Group 4 (G4), stool (Stool). Labels indicate genera at average relative abundance ≥2% in at least one body site. The remaining genera were binned together in each phylum as 'other' along with the fraction of reads that could not be assigned at the genus level as 'unclassified' (uncl.). See Additional file 1 for detailed values. **(b) **Circular cladogram reporting taxa consistently differential among the body habitats in at least one group detected using LEfSe. Colors indicate the group in which each differential clade was most abundant. See Additional file 3 for the detailed list of taxa whose representation was statistically different among the groups. The representation is based on RDP phylogenetic hierarchy.

At all phylogenetic levels, from phylum to genus, we identified four groups of body habitats that maintain a distinct pattern of numerically dominant bacterial taxa as profiled using the 16S rRNA gene (Figure 1a), as classified by the Ribosome Database Project (RDP) [29]. While only two phyla, the Firmicutes and Bacteroidetes, dominated the communities of all ten sites, their proportions and that of nearly all taxa in the sampled body habitats form groups as follows: Group 1, buccal mucosa, keratinized gingiva, and hard palate; Group 2, saliva, tongue, tonsils, and throat (back wall of oropharynx); Group 3, sub- and supra-gingival plaque; and Group 4, stool. The microbiota of Group 1 consisted mostly of Firmicutes followed in decreasing order of relative abundance by Proteobacteria, Bacteroidetes and either Actinobacteria or Fusobacteria (Figure 1a; Additional file 1). In comparison, Group 2 had a decreased relative abundance of Firmicutes and increased levels of four phyla: Bacteroidetes, Fusobacteria, Actinobacteria and TM7. Group 3, which consisted of both tooth plaque sites, had a further decrease in Firmicutes compared to Groups 1 and 2, with a marked increase in the relative abundance of Actinobacteria. Finally, the stool microbiota (Group 4) consisted of mostly Bacteroidetes (over 60%) followed by Firmicutes, with very low relative abundances of Proteobacteria and Actinobacteria, and less than 0.01% of Fusobacteria (Additional file 1).

These dramatic differences occurred consistently throughout the cohort, with closely juxtaposed body sites (for example, tongue dorsum (Group 2) and hard palate (Group 1)) possessing strikingly different microbial community structure even when considering the phylum level alone and independently of the structure of the tissue (Additional file 2). This supports strong local selective pressure on community membership even in the absence of disease, and these differences reach to the genus level (Figure 1a). In terms of genera, Group 1 was characterized by a very high relative abundance of *Streptococcus*, while Group 4 contained predominantly *Bacteroides*. In contrast, Groups 2 and 3, rather than having a single genus present at such high relative abundance, were characterized by a more even distribution of the most abundant genera. *Streptococcus*, *Veillonella*, *Prevotella*, *Neisseria*, *Fusobacterium*, *Actinomyces *and *Leptotrichia *were each present over 2% on average in Group 2. These seven genera plus *Corynebacterium*, *Capnocytophaga*, *Rothia *and *Porphyromonas *accounted for genera present at more than 2% in Group 3 (Figure 1a; Additional file 1).

Examining clade abundances at all taxonomic levels, we used the LEfSe (LDA Effect Size) system for biomarker discovery [30] to determine statistically significant biomarkers among these four groups within the digestive tract. These included both high and low abundance clades that significantly and consistently varied in abundance among and within body habitats, for example, in the three oral groups (Figure 1b; Additional file 3). For example, both the phylum Actinobacteria and individual taxa within the Actinomycetales were consistently more abundant on the tooth surfaces in Group 3 (Figure 1b; Additional file 3). When comparing Group 1 against the other three groups (a slightly more stringent setting than comparing all groups against each other as in Figure 1b and Additional file 1) two genera from the Firmicutes were identified as genus-level biomarkers: *Streptococcus*, from the Streptococcaceae (mean 47 ± 18% abundance in Group 1), and *Gemella*, from the Staphylococcaceae (mean 5.2 ± 5.1% abundance in Group 1) (Additional file 1). Although the Firmicutes phylum as a whole was most differentially abundant in Group 1, more specific taxa within the Firmicutes were detected as biomarkers for Groups 2 and 4 (Figure 1b; Additional file 3). For example, in Group 2, biomarkers, when compared to the other three groups, included *Oribacterium *and *Catonella*, members of the Lachnospiraceae, and *Veillonella*, a member of the Veillonellaceae (all Clostridia). The abundances of *Veillonella *and *Prevotella *overall were comparable in Group 2 (10.2 ± 5.4% versus 11.6 ± 7.3%, respectively), and both were identified as differentially abundant in this group. The other genus-level biomarkers for Group 2 detected at >2% were *Porphyromonas *(3.8 ± 4.2%) and *Neisseria *(6.6 ± 7.6%) (Additional file 1). Several genus-level biomarkers for Group 4 (stool) were also Firmicutes, mostly from the families Lachnospiraceae and Ruminococcaceae (Figure 1b; Additional file 3). These results support the overall consistency of the different microbial populations characterizing each of the four groups, and they also emphasize the need to take multiple levels of phylogenetic specificity into account when performing any analysis of the microbiome. Phylum relative abundances differentiated very distinct body habitats. As additionaly discussed below, these differences were reflected at the genus level within each body site in the healthy adult human.

The four observed groups differed significantly not only based on their specific microbial compositions, but also by several ecological summary statistics. Most strikingly, after comparing every pair of samples using the Bray-Curtis measure of beta diversity [31], within-group distance was very significantly lower (greater similarity) than between-group distance (lower similarity) for all four groups (Additional file 4; Table 1; all *P *< 10^-20^). The coarse level of species richness measurement offered by phylotype data did not distinguish strongly among any body habitats, but evenness and the resulting within-community alpha diversity ranged widely among groups as measured by the inverse Simpson index [32] (Additional file 5). For example, the Group 1 body sites together averaged below a relative diversity of 5.3, Group 2 ranged from 7.3 ± 3.0 (tonsils) to 10.6 ± 3.1 (saliva), the plaques in Group 3 had average diversities of 9.6 ± 3.1 and 9.8 ± 3.0, and Group 4 (stool) declined to a mean of 4.6 ± 2.9. The lower diversities in Group 1 are largely an effect of *Streptococcus *abundance, and likewise the gut microbiota's diversity is lowered by the prevalence of the *Bacteroides *in these data (both detailed above and below). These differences are highly statistically significant (for example, Group 1 versus 2 *P *< 1e-50 by *t*-test) and provide evidence in support of the four-group distinction at the levels of both individual bacterial clade and overall ecological structure.

**Table 1:** Community structure similarity is higher for samples in the same digestive tract group than for samples in different groups or outside the digestive tract

	Digestive tract groups				Non-digestive
	**G1**	**G2**	**G3**	**G4**	**tract samples**

**G1**	**0.58 ± 0.14**	0.43 ± 0.17	0.32 ± 0.13	0.02 ± 0.03	0.04 ± 0.06
**G2**	0.43 ± 0.17	**0.51 ± 0.14**	0.39 ± 0.11	0.05 ± 0.05	0.04 ± 0.06
**G3**	0.32 ± 0.13	0.39 ± 0.11	**0.49 ± 0.14**	0.03 ± 0.04	0.07 ± 0.08
**G4**	0.02 ± 0.03	0.05 ± 0.05	0.03 ± 0.04	**0.53 ± 0.17**	0.03 ± 0.07
**Non-digestive tract**	0.04 ± 0.06	0.04 ± 0.06	0.07 ± 0.08	0.03 ± 0.07	**0.29 ± 0.31**

Average Bray-Curtis index and standard deviations are reported for samples in each of the four digestive tract groups and body sites outside of the digestive tract. In bold are highlighted the within group similarity values that are statistically significantly higher (*t*-test *P *< 1e-20) than any between-group distances. The body sites outside of the digestive tract included three vaginal sites (posterior fornix, mid-vagina, vaginal introitus), the nasal cavity (anterior nares), and two skin sites (antecubital fossae and retroauricular crease).

### Phyla typically identified with environmental communities are part of the natural microbiota of healthy humans

**Figure 2:**
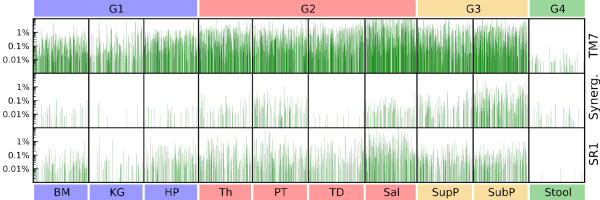
**Noticeable relative abundance and variability of TM7, Synergistetes, and SR1 per body habitat**. Representation of the relative abundances of the phyla TM7, Synergistetes (Synerg.), and SR1 among the subject population, expressed as percentage on a log scale (left). The high relative abundances of members of these phyla among the subjects, in particular for TM7, indicate a potential role in eubiosis. The body habitats and groups are labeled as in Figure 1.

Bacterial phyla originally thought to be exclusively environmental have recently been observed to possess human host-associated membership [33-36]. This phenomenon was widely observed within this normal population. The phylum TM7 was highly prevalent, detected in at least one sampling site of the upper digestive tract of 85% of subjects and in the stool of 13.6% of the subjects (Additional file 6). The phyla SR1 and Synergistetes were present in at least one upper digestive tract site of 65.3% and 58.5% of the subjects and in the stool of 1.4% and 8.8% of the subjects, respectively. The phylum Verrucomicrobia, represented mainly by the genus *Akkermansia *[35], and the phylum Lentisphaerae, represented by the genus *Victivallis *[34], were present in the lower digestive tract of 41.5% and 15.0% of the subjects and in the upper digestive tract of 13.6% and 1.4% of the subjects. TM7 bacteria accounted for a mean of 3.1 ± 5.7% of the saliva population and 0.6 ± 1.2% of the bacteria found in plaque communities (Figures 1a and 2; Additional file 1). The SR1 phylum was also most abundant in saliva (mean 0.4 ± 1.2%), and both TM7 and SR1 phyla were found at trace amounts in stool. While these phyla were varyingly prevalent (Figure 2), they occurred near-uniformly at low but significantly non-zero abundances, which highlights their lack of detection in smaller studies without deep high-throughput sequencing.

### Genera characterized by pathogenic members and thus associated with disease were prevalent at low abundance in the normal human microbiota

**Figure 3:**
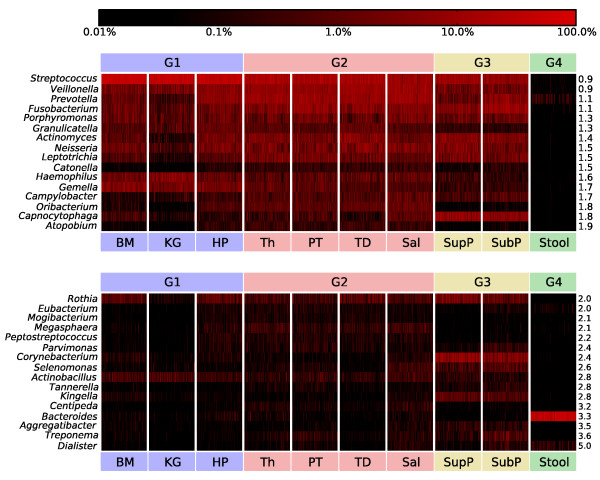
**Most microbes in the digestive tract communities vary widely in relative abundance among body habitats and individuals**. Genera with the lowest (top) to highest (bottom) variability among samples spanning all ten body sites, with coefficients of variation reported numerically (right column) and relative abundance colored on a log scale. The scale bar shows the color-coding of the average relative abundance expressed as percentage, from low (black) to high (red). All genera present >0.001% in at least half of the samples are reported. Prevotella, Veillonella, and Streptococcus are least variable across both body sites and individuals.

**Figure 4:**
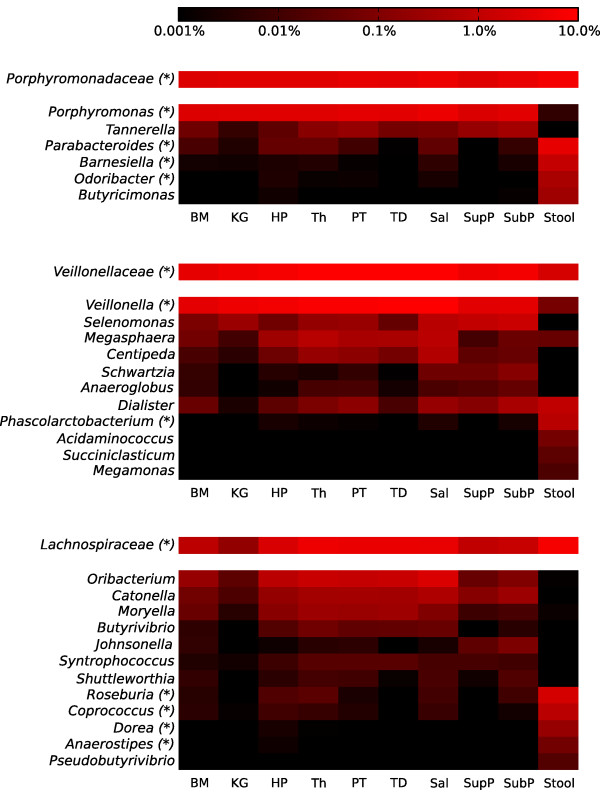
**Genera within the Porphyromonadaceae, Veillonellaceae and Lachnospiraceae families are differentially abundant across microbial communities between the upper and lower digestive tract**. These three families were detected among all ten digestive body habitats, but genera within them showed varying patterns of niche specialization to sites along the digestive tract. All genera with at least 0.001% abundance in at least one body site are reported here. Clades showing a statistically significant difference (by LEfSe) specifically between oral and stool samples are indiocated with asterisks. Abundances are reported on a log scale as averages. The scale bar shows the color-coding of the average relative abundance expressed as percentage, from low (black) to high (red). The Porphyromonadaceae family is interesting in that its average abundances are higher in the gut than in the oral body habitats, but specific genera within the family diverge: Tannerella and Porphyromonas are predominantly present in the oral cavity, whereas Parabacteroides, Barnesiella, Odoribacter and Butyricimonas show higher relative abundances in the gut. BM, buccal mucosa; KG, keratinized gingiva; HP, hard palate; Th, throat; PT, palatine tonsils; TD, tongue dorsum; Sal, saliva; SupP, supraginval; SubP, subgingival plaques.

Clades populated with known bacterial oral pathogens were well represented in this reference adult cohort, typically with moderate to high population penetrance but low relative abundance in each individual. Among the Spirochetes, Treponema species are associated with periodontal and endodontic diseases [37,38] and were present in at least one of the upper digestive tract sites of 96% of this disease-free population (and in all the nine oral sites of 6.8%). Treponema had a variable relative abundance among the oral body habitats, with highest representation in the subgingival biofilm (mean 2.2 ± 4.1%) and non-zero abundances in several other sites, for example, palatine tonsils (Figure 3; Additional file 1). In contrast, a minority of stool samples (3.4%) contained trace levels of Treponema. The previously published rarity and specificity of Brachyspira to the gut was confirmed by its detectable presence in only one stool sample (226 stool samples in total; Additional file 7) and absence from all the upper digestive tract sites (1,879 samples; Additional file 7). Other periodontal pathogens were lower in abundance. Aggregatibacter were found mostly along the tooth surfaces (Group 3; mean 0.4 ± 0.7/0.8% from supra- and sub-gingival biofilms), and Megasphaera were found mostly in Group 2 (from mean 0.4 ± 0.6% in the tonsils and tongue dorsum to 0.8 ± 0.9% in saliva). Bifidobacteriaceae, implicated in the formation of caries [39,40], were very rare at all oral sites (means <0.03%), but possessed high prevalence (40.8%). In the stool, the genus Bifidobacterium was most represented with a low mean relative abundance of 0.08 ± 0.3%. The low abundance of Bifidobacteriaceae in the oral cavity may be a reflection of the lack of carious lesions in this healthy subject population. Porphyromonas , which includes Porphyromonas gingivalis (one of the most studied oral pathogens) and non-pathogeneic strains, was present in the upper digestive tract of all the subjects (mean 3.0 ± 3.8%, 3.8 ± 4.2%, and 3.0 ± 3.5% in the three oral groups, respectively) and in 25% of the lower digestive tract samples, though in very low abundance in the stool (Additional file 1). Tannerella, thought to incur similar host phenotypes, was present in the upper digestive tract of 97.3% and in the stool of 3.4% of the subjects. Both genera, Porphyromonas and Tannerella, were almost uniquely distributed in average abundance among individual body sites within the oral cavity, whereas the other relevant genera in the family Porphyromonadaceae (Parabacteroides , Barnesiella , Odoribacter, and Butyricimonas) predominantly colonize the stool (Figure 4).

Genera that include important human pathogens colonizing the throat/tonsils - *Streptococcus pneumoniae*, *Streptococcus pyogenes*, *Neisseria meningitidis*, and *Haemophilus influenzae *- were all well represented in the microbiota of the upper digestive tract sites (Figure 1a; Additional file 6). The known difficulty of performing species-level identification from 16S rRNA pyrosequencing experiments [41] precluded the determination of prevalence for these specific pathogens in this cohort. The genus *Moraxella*, which includes the common sinus pathogen *Moraxella catarrhalis*, was detected in the upper digestive tract microbiota at low relative abundance, reaching a mean >0.5% only in the throat (Additional file 1). Interestingly, the high standard deviation (4.7%) of the relative abundances of *Moraxella *in the throat suggested variable colonization within this population.

In the lower intestinal tract, genera containing known pathogens were typically low in both prevalence and relative abundance. *Helicobacter*, implicated in gastrointestinal diseases, appeared in only 1.4% of stool samples in trace amounts while studies of *Helicobacter pylori *stool antigen prevalence in healthy European adults ranged up to 33% [42]. Enterobacteriaceae abundances were uneven among individuals in the gut and within each individual among body sites, with the most abundant genus being the *Escherichia*/*Shigella *complex (mean 0.1 ± 0.67%), which was detected in 33% of stool samples. Finally, *Faecalibacterium*, a genus of considerable interest due to its observed decrease in abundance in active Crohn's disease [43-46], was highly represented in the stool (98% of subjects and mean 4.6 ± 5.2%) but present only at trace levels in the oral cavity (always below 0.05%), suggesting that it may be adapted to a very specific niche within the gut.

### Comparison of microbial communities from the two tooth surface-associated sites

**Figure 5:**
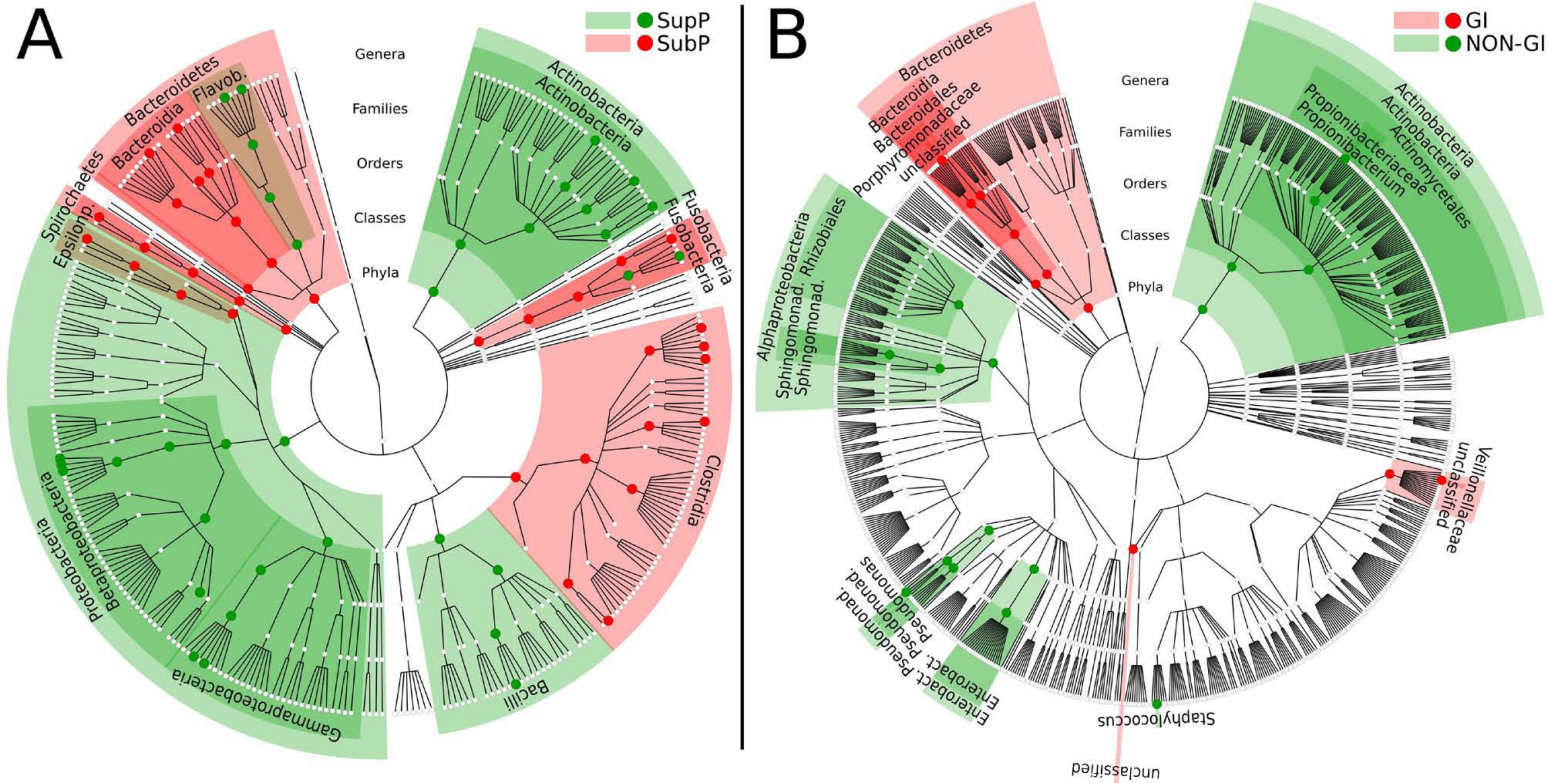
**Niche specialization is widespread throughout the digestive tract even among adjacent body habitats**. **(a) **Circular cladogram based on the RDP Taxonomy [29] reporting taxa significantly more abundant in supragingival (red) and subgingival plaque (green) and demonstrating the extensive specialization even at these highly related sites. At the class level, Actinobacteria, Bacilli, Gamma-proteobacteria, Beta-proteobacteria, and Flavobacteria are characteristic of the supragingival plaque, whereas Fusobacteria, Clostridia, Epsilon-proteobacteria, Spirochaetes, Bacteroidia, and unclassified Bacteroidetes are biomarkers for the subgingival plaque. **(b) **Circular cladogram comparing the digestive tract (red, GI) with non-mucosal body habitats (green, NON-GI: comprising samples from the anterior nares, and from the bilateral skin sites, antecubital fossae, and retroauricular creases). Only a few clades are detected as differentially present and abundant throughout the entire digestive tract, as the high degree of specialization and community variability at each body site prevents any individual community member from being representative of all ten body habitats. BM, buccal mucosa; TD, tongue dorsum; SupP, supraginval.

Within the oral cavity, the Group 3 sub- and supra-gingival plaque bacterial communities were most distinct and differed strongly from the other body sites, but further differences characterized each of these two sites individually. The tooth surface adjacent to the soft gingival tissues specifically comprises two distinct ecological niches, supragingival, and subgingival (Additional file 8). The supragingival region sits above the gingival margin, exposed to the oral cavity, bathed in saliva and exposed to ingested substances; the subgingival region is bathed in a serum transudate that flows from the base of the crevice outward to the oral cavity. A key known physiological difference between these two regions is the lower redox potential found subgingivally [47]. Correspondingly, we observed differences in the non-diseased plaque biofilm communities from these two regions distinguished by proportional shifts consistent with these physiological distinctions (Figure 5a; Additional file 1). Shifts at the phylum level were driven by subgingival increases in the obligate anaerobic genera Fusobacterium, Prevotella, and Treponema, and by lesser relative abundances of Dialister, Eubacterium, Selenomonas, and Parvimonas. In contrast, groups significantly increased in the supragingival plaque consisted predominantly of facultative anaerobic genera, including Streptococcus, Capnocytophaga, Neisseria, Haemophilus, Leptotrichia, Actinomyces, Rothia, Corynebacterium, and Kingella (Figure 1a; Additional file 1). This suggests that along these tooth surfaces, where direct bacterial interaction with host cells is diminished, oxygen availability - an environmental factor - may be a major driver of community composition.

### The oropharyngeal microbiota lacked abundant site-specific bacteria across all samples when compared to the mouth

The pharynx is the site of carriage of a number of important bacterial pathogens that impact both healthy and immunocompromised individuals. LEfSe analysis of all samples did not identify any genus-level organisms characteristic of the microbiome of the throat and/or tonsils consistently present above our limit of detection. For example, when throat and tonsil samples were compared to the mouth sites, the genera *Butyrivibrio *and *Mogibacterium *(both from the phylum Firmicutes) were identified as differentially abundant, but both were present at only low levels (mean 0.057 ± 0.09%, and 0.188 ± 0.316%, respectively, corresponding to only approximately 1 to 5 sequences/sample; Additional file 1). The palatine tonsils, located in the oropharynx, are unique among the sites sampled in this study as the only lymphoid tissue. However, the genus-level tonsil-specific biomarker when compared to the mouth, *Peptococcus*, was again present at very low relative abundance (mean 0.049 ± 0.079%; Additional file 1). This lack of throat- or tonsil-specific biomarkers among bacterial taxa with a relative abundance >1% likely reflects the similarity of the microbiome of these two oropharyngeal sites with those of the tongue dorsum and saliva (Group 2 in Figure 1) despite their differences in tissue type (Additional file 2). This observation is supported by the comparison of the complete Group 2 with all other groups, which revealed distinct and abundant biomarkers as discussed above (Figure 1b; Additional file 3). Microbiota composition and the path of swallowed saliva suggest a potential role of saliva as one of the host factors influencing microbiota of Group 2.

### No genus-level microbial biomarkers characterize the entire digestive tract microbiota as contrasted with non-mucosal body habitats

After analyzing the microbiota of body habitats within the digestive tract, we next asked if there were bacteria whose differential abundance characterized the digestive tract as a whole. The non-mucosal sites sampled in the HMP included anterior nares, both post-auricular creases (crease behind the ear), and both antecubital fossae (inner elbow crease). ***Propionibacterium***, ***Staphylococcus***, ***and Pseudomonas ***were identified as biomarkers for the non-mucosal sites, based on a LEfSe analysis of all ten digestive tract sites versus the non-mucosal sites (Figure 5b). However, no genus-level biomarkers were identified as uniquely present throughout the digestive tract microbiota. The unclassified Veillonellaceae and Porphyromonadaceae (Figure 5b) are unlikely to be true biomarkers due to their low representation. Further analysis was impaired by the lack of reference sequences for them within RDP. Members of Veillonellaceae and Porphyromonadaceae families were much less abundant at non-mucosal sites, and were essentially absent from the HMP vaginal samples, suggesting that their adaptation is to the digestive tract mucosa rather than mucosal surfaces in general.

### Bacterial families common throughout the digestive tract possess variable distributions of genera distinct to upper and lower sites

Bacterial genera membership overlap in the same subject between oral and stool samples was limited when considered if present in at least 45% of the subjects. It included Bacteroides, Faecalibacterium, Parabacteroides, Eubacterium, Alistipes, Dialister, Streptococcus, Prevotella, Roseburia, Coprococcus, Veillonella, Oscilibacter, and yet-to-be-classified genera from a subset of families (Additional file 6). Interestingly, the presence of genera in a large portion of subjects was not related to a stable relative abundance in the microbial communities, as Bacteroides and Dialaster were among the four most variable genera among subjects. In contrast, Prevotella, Veillonella and Streptococcus were the genera with the most consistent presence in the subject population (Figure 3). The importance of Lachnospiraceae, Veillonellaceae, and Porphyromonadaceae families in the healthy digestive tract microbiome was indicated by their relative abundance among all body habitats and among subjects (Figures 1 and 4; Additional files 1 and 6). Bacteria of the Lachnospiraceae and Veillonellaceae families specifically were present in all subjects' oral cavities and stools (Additional file 6). Porphyromonadaceae were present in the oral cavity of all subjects and the stool of 95.9% of subjects (Additional file 6), although their relative abundance of member genera varied by body habitat (Figure 4). Porphyromonas was present primarily in the oral sites, while Parabacteroides, Barnesiella, Odoribacter and Butyricinomonas were predominant in the stool (Figure 4). The significance of this variation in genus distribution was confirmed by LEfSe conmparisons of the upper (oral) and lower (stool) digestive tract sites (Additional file 3). In contrast, Tannerella (Figure 4) was present in most oral sites, but due to a lower relative abundance specifically in the keratinized gingiva, it was not found to be statistically signicant between the oral and gut sites. The pattern of variable genus distribution between the upper and lower parts of the digestive tract holds for the Lachnospiraceae and Veillonellaceae as well (Figure 4), again suggesting a pattern of niche specialization among human body habitats extending from the bacterial family level down to specific genera.

### Differential representation of microbial metabolic function among body sites using reconstruction from whole shotgun sequencing

**Figure 6:**
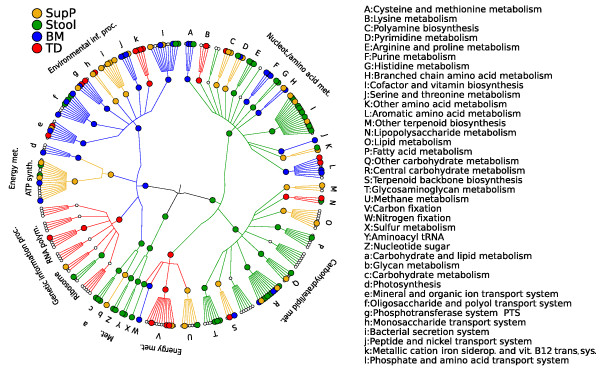
**Functional characterization of the digestive microbiota based on metabolic pathway abundances in the buccal mucosa, supragingival plaque, tongue dorsum, and stool from metagenomic shotgun sequencing**. Cladogram represents the KEGG BRITE functional hierarchy, with the outermost circles representing individual metabolic modules and the innermost very broad functional categories. Pathways coloration denotes modules showing significant differential abundances in at least one of the four body habitats. Metabolic profiling was performed with HUMAnN [48], revealing a much lower degree of variability among individuals and significant specifity of many pathways' relative abundance to individual body habitats. In particular, sugar transport and metabolism varies at each of the four habitats with metagenomic data, as does iron uptake and utilization.

In addition to relative abundances of bacterial organisms based on 16S rRNA genes, we examined the abundances of microbial metabolic pathways as profiled from metagenomic shotgun sequencing of a subset of the available body habitats [48]. These data from the HMP included one body site within each of the four digestive tract groups: the buccal mucosa (Group 1), the tongue dorsum (Group 2), the supragingival plaque (Group 3) and the stool (Group 4). The data analyzed below include the relative abundances of individual enzyme families (Kyoto Encyclopedia of Genes and Genomes (KEGG) Orthologous groups (KOs) [49]) and of complete metabolic modules (KMods) (Figure 6; Additional file 9).

Bacterial cells use a wide variety of aerobic or anaerobic degradation pathways as energy sources, and this was most evident in the differences in relative abundance of specific sugar transporters when comparing the oral sites to the gut. PTS transporters for small sugars were most abundant in the oral cavity and were represented for monosaccharides by mannose (M00276) and fructose (M00304) transporters, as well as the transporter of galactosamine (M00287), derived from the breakdown of sugar-decorated glycoproteins. The supragingival plaque microbiome was enriched for threhalose (M00270, M00204), alpha-glucosides (M00201, M00200), and cellobiose (M00206) transport; in contrast, the stool microbiome was enriched for the transport of lactose/arabinose (M00199) and oligogalacturonide (M00202), and for the degradation of the larger dermatan (M00076), chondroitin (M00077) and heparin (M00078) sulfate polysaccharides. Surprisingly, while anaerobiosis-related pathways were expected throughout the digestive tract, putrescine transporters in particular were most represented in the oral cavity (M00193, M00300). This is of potential interest as concurrent production and import of putrescine is a delicate balance, and excess putrescine is one source of halitosis [50].

Consistent with what is known about the function of the colonic gut bacteria, we observed several prominent enzymes and metabolic pathways most abundantly in the stool metagenome. For instance, β-glucosidase (K05349) was specifically abundant in the gut microbiota and not at oral sites; this enzyme is critical in the pathway of cellulose breakdown to β-D-glucose. Concomitantly, given that the Embden-Meyerhoff pathway is also known to be the major route for glucose metabolism to pyruvate in the colon, the highly associated glycolysis pathway module (M00001) was also significantly enriched in stool [51]. This finding is further in agreement with the 16S rRNA gene sequencing data, which included prevalent *Ruminococcus *in stool that are important colonizers of plant-derived material in the gut and possess cellulolytic activity [52]. The stool bacteria were also uniquely associated with pathways for ammonia (M00028, urea cycle, and M00029, ornithine biosynthesis) and methane (M00356 and M00357, both methanogenesis) production; the prominence of these enzymes is consonant with the colonic microbiome as a significant source of ammonia production. In fact, targeting the colonic microbiome with antibiotics has been shown to be a successful therapy in acquired diseases of hyperammonemia such as encephalopathy complicating hepatic insufficiency [53]. Relatedly, compared to upper digestive tract sites, there was very high abundance of a specific multiple antibiotic resistance protein (K05595) and association with the pyruvate:ferredoxin oxidoreductase pathway, which, due to its role in converson of metronidazole to its active nitroso form, can also determine sensitivity to this antibiotic. These potential pathogenically linked behaviors are of course in addition to the expected colonic bacterial activities detected for producing energy from undigested cellulose, nitrogen-containing compounds, and vitamins and cofactors important in support of basic metabolic pathways.

Although HMP protocols were optimized for bacterial sequences, shotgun sequencing also provides an initial means of assessing the community structure of non-16S assayable microbes. As reported in Additional file 10, the fractions of Archaea (0.04% in the stool; below the detection threshold in the oral cavity) and small eukaryotes (0.34% in the buccal mucosal; <0.1% in the other body sites) detected here proved to be very small. Although this may be due in part to the HMP's specific DNA handling protocols [10], this suggests that 16S rRNA gene-based community surveys provide an accurate overview of these digestive-tract associated microbial communities. Likewise, ribosomal and shotgun sequencing in the HMP cohort have been compared elsewhere and provide consistent quantitative estimates of genus-level abundances [54] without systematic phylum-level biases.

### Integration of gene/pathway abundances from metagenomic data and bacterial clades based on 16S rRNA gene data

A subset of the HMP's microbiome samples was assayed with both shotgun metagenomic and 16S rRNA gene sequencing. This allowed us to assess co-variation of microbial abundances with inferred metabolic pathways. Strong correlations between the abundances of bacterial clades (from 16S rRNA data) and gene or pathway abundances (from metagenomic data) in some cases clearly highlighted genes carried by these organisms, and in others denoted less clear pangenomic elements or metabolic dependencies. An example of the former was the arabinofuranosyltransferase genes *aftA *and *aftB *(K13686 and K13687). These genes were only present in the tooth surface habitats and are known to be encoded by *Corynebacterium*, a biomarker of the plaques as discussed above. This was confirmed by the genes' strong association with *Corynebacterium *clades in these data (Spearman correlation 0.76, *P*-value <1e-15; Additional file 11). Archaea were not included in our analysis, as these were not detectable by 16S rRNA gene sequencing (due to lack of the conserved sequence in the primers used) and were poorly represented in the shotgun sequencing data.

The acquisition and export of metals for bacterial homeostasis and for pathogenicity is ubiquitous throughout the human microbiota, with iron being most generally necessary. Iron transporters were widely distributed among the microbiota of all four body sites, but again, the specific mechanisms of iron uptake and sequestration differed as needed for niche specialization. One use of the iron is its incorporation in porphyrin, and there was a wide distribution of cytochrome c heme lyase (K01764), which appeared to be ubiquitous and was not strongly associated with individual organisms. Conversely, uroporphyrinogen synthase (K01719) occurred at higher relative abundance in stool, inversely associated with members of the Clostridiales (Spearman correlation -0.79, *P*-value <1e-15; Additional file 11). This can be contrasted to protoporphyrinogen oxidase (K00231) in the oral cavity, which is potentially linked to the *Prevotella *enrichment (Spearman correlation 0.71, *P*-value <1e-15). Within the oral cavity specifically, coproporphyrinogen oxidase (K00228) and protoporphyrinogen oxidase (K00231) were both more abundant on the tongue and in supragingival plaque than on the buccal mucosa, expected to be linked to the increased relative abundance of *Porphyromonas *and *Prevotella *on those surfaces [55] (Figure 1; Additional file 11).

Metal export and utilization were likewise ubiquitous throughout the microbiota, but differed in the prevalence of specific mechanisms. Most genes encoding exporters needed for heme tolerance [56], such as MtrCDE (K00579, K00580) and HrtAB (K09814, K09813), were present at low levels throughout the digestive tract, although MtrCDE was somewhat enriched in the more anaerobic habitats, stool and plaques. None were significantly associated with specific organisms in these data. The gene encoding hemerithryn (K07216) was detected at multiple body sites but was highly enriched in stool. This enzyme for iron utilization is most often found in members of the Methylococcaceae family [57], but these were again not detectable in this study due to their absence from the RDP 16S rRNA database (see Materials and methods). Intriguingly, the hemerithryn (K07216) gene consistently associated with members of the Clostridiales when present in the gut (Spearman correlation 0.72, *P*-value <1e-15; Additional file 11). Finally, other metals, including copper and zinc, are also both necessary co-factors and potential toxins, and remediation pathways and transporters for both were observed consistently (copper resistance K07245; copper homeostasis K06201, K06079; zinc resistance K07803; and also many other metal transporters).

Although recent work has provided extensive insights into the mechanisms of bacterial interaction with the host immune system in the gut, much less is known about the relationship of the microbiota with host immunity for other body habitats and cell types. Two pathways observed in both the upper and lower digestive tracts and known to be involved in immunomodulation were hydrogen (H_2_) and hydrogen sulfide (H_2_S) production. Hydrogen production has been shown to be an important byproduct of acetogenic bacteria and also has an anti-inflammatory activity [58]. Enzymes both for utilization (for example, CoM methyltransferase, K14082) and for production (for example, hydrogenase-4, K12136) of hydrogen were identified specifically in the oral cavity (nearly completely absent from the gut), with potential bacterial contributors including *Veillonella *and *Selenomonas *species genomically [59] and, in one of the strongest links between genes and organisms in these data, an unclassified Pasteurellaceae clade in the oral cavity (Spearman correlation for K12136 >0.78, *P*-value <1e-15 in supragingival placque and tongue dorsum).

Hydrogen sulfide gas is involved in regulation of the host response at low concentrations and in host-cell toxicity and inhibition of short chain fatty acid production, specifically in the colon, at high concentrations [60-65]. H_2_S may thus serve different purposes among the distinct bacterial communities of the digestive tract. The potential for its production was particularly enriched in stool (for example, by cystathione-beta-lyase, K14155), and somewhat enriched in the more anaerobic habitats, stool and plaques (for example, by methionine-gamma-lyase, K01761). A possible role in host-cell toxicity was strongly suggested by K01761's close association with the *Treponema *and *Fusobacterium *genera in plaque (Spearman correlation 0.74 and 0.82, respectively, *P*-values <1e-15), both of which include members specifically associated with periodontal disease (Additional file 11). These genes were again, however, present at low levels among all body sites analyzed here, consistent with a low-level immunomodulatory role for H_2_S throughout the digestive tract.

## Discussion

The large reference population of the HMP has provided, to our knowledge, the first opportunity for a comprehensive description of the human gastrointestinal microbiota, focused here on the bacterial composition and function of ten independently sampled body habitats throughout the digestive tract. Using taxonomically binned 16S rRNA gene sequences, we identified the representation and relative abundance of organisms in 2,105 samples. We used the LEfSe system for metagenomic biomarker discovery to identify clades at all taxonomic levels whose distribution varied among four classes of body habitats, and which included rare clades not expected as commensals in the human microbiome. We also observed prevalent but low abundance of genera characterized by common pathogenic species, even in this asymptomatic reference population. Finally, we performed a complementary analysis of the metabolic modules and enzymes detected in a subset of these body sites, revealing strong variation in sugar and metal utilization among the digestive tract communities.

Four distinct groups were delineated among the microbial communities from the digestive tract sites. The groups were rooted in the ratio of the relative abundances of the two major phyla, Firmicutes and Bacteroidetes (Figure 1a), and the differences extended to the genus level. In the absence of disease, these groupings suggest that it might be possible to sample one representative site from each group in future studies as a strategy to decrease sequencing costs. For example, the buccal mucosa (Group 1), tongue dorsum (Group 2), supragingival plaque (Group 3) and stool (Group 4) could be used to represent all ten sites examined here. Samples from the suggested body habitats can be obtained with minimal discomfort and risk to participants, and are likely to provide the biomass needed to yield sufficient DNA for community whole genome shotgun analysis. Since the current study includes only healthy subjects, however, additional validation would be required to investigate pre-disease and disease states at targeted sites for both local and systemic diseases.

The oral microbiome as revealed in this investigation was generally consistent with earlier studies [11,13,14,22,66,67]. Firmicutes largely dominated the microbial communities on oral tissue surfaces and in saliva. Dental plaque taxa were more evenly distributed, dominated by Firmicutes, Bacteriodetes, Actinobacteria, Proteobacteria and Fusobacteria. The differences in the plaque communities relative to oral tissue sites are likely driven by the ability of the microbial community to accumulate on the non-shedding tooth surface and the physiological status relative to oxygen distribution in the resulting biofilm. *Porphyromonas*, *Tannerella *and *Treponema*, genera consisting of recognized pathogens in periodontal diseases, were highly prevalent. The presence of these genera in greater than 95% of individuals in this non-diseased population provides strong evidence that they are part of the commensal oral microbiome. These data suggest, rather than a complete absence of pathogenic organisms from the normal microbiota, the possibility of low-level carriage of potential pathogens [68-70].

The stool microbiota was distinguished from the microbiota of the upper digestive tract sites (Figure 1a), as expected, and set apart by a high abundance of Bacteroidetes. A notable difference in the composition of the stool microbiome of the HMP dataset compared to existing 16S rRNA gene profiles is the increased ratio of Bacteroidetes (>60% of the sequences) to Firmicutes (≤30% of the sequences). Many previous studies of adult American populations have observed the reverse, a preponderance of Firmicutes [15,71-73], and similar observations have been reported in geographically diverse populations [74,75] and in infant gut microbiome colonization investigations [76]. It should be noted that all HMP gut communities were assayed from stool samples, which may differ extensively from colonic biopsies. For example, using endoscopic biopsies from just two subjects, Wang *et al*. [77] reported 49% of 16S rRNA gene clones were from the Firmicutes and 27.7% were from Bacteroidetes. However, even this distinction is unclear, as a study of 16S rRNA sequences from regional gut biopsies and spontaneously passed stool involving three subjects similarly showed the majority of phylotypes belonged to Firmicutes (76%) compared to 16% for Bacteroidetes [15]. In a study of stool from 154 adult women (twins and their mothers), Firmicutes had a mean relative abundance of >60% using several different methods to assess the 16S rRNA gene content of stool [24]. Finally, a recently published study of fecal microbiota in 161 older subjects (≥65 years) corroborate our findings, namely a Bacteroidetes-dominant distribution (57%) compared to Firmicutes (40%) [26]. The difference in the Firmicutes:Bacteroides ratio in stool samples analyzed by 16S rRNA composition was confirmed by whole genome shotgun data from the same samples in the HMP dataset [54]. While it is possible that these differences are linked to any of geographic location, host genetics, or differences in technical procedures, further study will be critical in explaining these apparently dramatic variations in gut microbiota composition in adults.

An estimated 10^11 ^bacterial cells per day flow from the mouth to the stomach [78,79]. Both cultivation and molecular techniques demonstrate an overlap in the oral, pharyngeal, esophageal and intestinal microbiomes [12,27,28,75,80-85]. It has thus been hypothesized that the oral microbiota might significantly contribute to distal digestive tract populations. Among HMP subjects, the genera *Bacteroides*, *Faecalibacterium*, *Parabacteroides*, *Eubacterium*, *Alistipes*, *Dialister*, *Streptococcus*, *Prevotella*, *Roseburia*, *Coprococcus*, *Veillonella*, and *Oscilibacter *were detected in both the oral cavity and stool in more than 45% of subjects. However, the short sequence reads did not permit species-level identification, leaving open both the possibility that there are distinct distributions of species of these common genera along the digestive tract, and the question of whether oral microbes seed distal sites below the stomach.

Based on the commonality of genera detected in the upper digestive tract, we postulate that saliva, via its impact on pH (as a buffer) and nutrient availability (high mucin content) [86], is a key driver of microbial composition in the habitats above the stomach. The epithelium is likely another key driver as most of the upper gastrointestinal mucosal surfaces share a common epithelial lining (nonkeratinized, stratified, squamous epithelium), with the exception of the keratinized gingiva, hard palate and parts of the tongue dorsum, which instead share a keratinized, stratified, squamous epithelium (Additional file 2). The upper digestive tract sites are also constantly exposed to both inhaled and ingested microbes. A substantial portion of the variability observed in the upper digestive tract tract microbiota might then be explained by interactions between the saliva, host cell type, and exogenous factors such as oxygen availability and oral intake.

In contrast to these potentially homogenizing effects, the throat, among the nine upper digestive tract sites sampled, is uniquely the recipient of small particles, including microbes, that are trapped in mucus and propelled by respiratory cilia up from the trachea and down from the nasal cavity *en route *to the stomach. This might impose an additional selective pressure on pharyngeal microbiota. However, no such effect was evident in the oropharynx, which segregated nicely into Group 2 with sites not exposed to the constant flow of respiratory tract mucus. Group 2, with the tongue, tonsils, throat and saliva, is a reminder of the important overlap between the upper segments of the digestive and respiratory tracts: the aerodigestive tract, which consists of the 'lips, mouth, tongue, nose, throat, vocal cords, and part of the esophagus and windpipe' [87]. Evidence suggests that the pool of microbes from Group 2, and other oral sites, contribute to colonization of the airways in disease. A few examples of this from the polymicrobial airway infections of cystic fibrosis follow: one of the earliest cystic fibrosis pulmonary pathogens is *Haemophilus influenzae*, a common colonizer of the upper aerodigestive tract [88]; members of the *Streptococcus milleri *group were recently implicated as cystic fibrosis pathogens [89], and are known colonizers of the oral cavity; and lastly, members of the oropharyngeal microbiome might modulate the virulence of the key cystic fibrosis pathogen *Pseudomonas *[90]. To explain microbial community structure throughout the aerodigestive tract and airways, one might speculatively extend the basic argument above, noting that the counterpart of saliva is mucus in regions not bathed by its flow, including sites sampled by the HMP but not investigated here (for example, the anterior nares) and habitats that require more invasive methods for sampling (for example, nasal cavity, nasopharynx, esophagus and airways).

Several 'environmental' phyla observed in human microbiota [33,91] appear to be strongly host-associated in this study. The Synergistetes phylum, for example, has only recently been described in detailed association with the human oral cavity [36,92], and is still considered potentially environmental due to its common occurrence in, for example, bioreactors [93,94]. Although completely absent from all ten sites in many individuals, it conversely comprised up to 10% of the community in some samples, and tended to recur at multiple body habitats within the same individual. This property - a dichotomy of apparent niches that includes specific and potentially stable occupation of human microbiome sites - can now be extended to TM7 and SR1 based on the HMP oral cavity data. As sequencing costs drop, deeper shotgun sequencing will provide access to such organisms with higher confidence, as most of those organisms are only known through their phylogenetically conserved genes.

## Conclusions

Analysis of the HMP dataset described here has provided a comprehensive characterization of the disease-free digestive tract microbiome, and will further serve as a foundation for the study of comparable disease-associated microbial communities. By surveying the HMP population, these results can be further integrated into other currently ongoing studies of the cohort's core microbiome [9] or enterotype structure [25], if any. The personalized nature of the digestive tract microbiota revealed here speaks to its potential as a therapeutic target or point of intervention in genomic medicine, particularly as future studies are able to additionally account for host genetic polymorphism. Few examples yet exist where the overall composition, relative abundances, or microbial proportions of a microbiome are conclusively causal in human disease. However, it is clear that disease states are often associated with a disruption of the microbial community, frequently resulting in one or a few pathogenic organisms emerging [95,96]. A classic example of this is the frequent ingestion of fermentable sugars that leads to increases in the mutans streptococci, etiological agents of dental caries [97]. Similarly, in the periodontal subgingival habitat, ecological shifts in redox potential facilitate the emergence of anaerobic pathogenic microbes such as the porphyromonads, which are prevalent but in low abundance in the non-diseased state [97,98]. It is likely that microbial biomarkers at one or more body habitats will eventually be found to be prognostic indicators of future disease status, and even this reference population could contain as-yet-undetected pre-disease states. We thus hope that this profile of the human microbiota will provide a reference for subsequent investigations of its role in the onset and alleviation of diseases along the human digestive tract.

## Materials and methods

### Population recruitment, sample collection, and DNA purification

Healthy adults 18 to 40 years old were recruited at two academic centers [10]. Fifteen and 18 body habitats were collected from enrolled males and females, respectively. The sites sampled included anterior nares, oropharynx (two specimens), oral cavity (seven specimens), skin (four specimens), stool, and vagina (three specimens per female) [10]. The Manual of Procedures and the Core Microbiome Sampling Protocol are available at the Data Analysis and Coordination Center for the HMP [99], as well as dbGaP [100]. Genomic DNA was isolated from the collected samples using the MO Bio PowerSoil DNA Isolation Kit (MO BIO laboratories, Inc., Carlsbad, California, USA) [10].

### Sequencing and binning of 16S rRNA genes and read processing

Detailed protocols used for 16S rRNA bacterial gene amplification and sequencing, using the 454 FLX Titanium platform and kits (Roche Diagnostic, Corp., Indianapolis, Indiana, USA), are available on the HMP Data Analysis and Coordination Center website [99], and are also described elsewhere [10]. In brief, sequences were processed using a data curation pipeline implemented in mothur [10,101] starting with quality trimmed for homopolymer runs and a minimum 50 bp window average of 35. Any sequences not aligning against the appropriate subset of the SILVA database [102] were removed, as were chimeric sequences. Resulting sequences were processed using a data curation pipeline implemented in mothur [10,101]. Remaining sequences were classified with the MSU RDP classifier v2.2 [29] using the taxonomy maintained at the RDP (RDP 10 database, version 6). Definition of a sequence's taxonomy was determined using a pseudobootstrap threshold of 80% [10].

### 16S rRNA gene dataset post-processing and quality control

A table of read counts from the 16S rRNA bacterial gene pipeline was created by summing clade counts from the three regions and was further processed for removing low-coverage samples. Firstly, those taxa not supported in the whole dataset by at least two sequences in at least two samples were removed. Then, the quality control procedure compared, for each sample, the read count of the most abundant taxon *t *and the highest abundance value that the same taxon *t *achieved in the entire dataset. If the former term of the comparison is <1% of the latter, the sample was discarded. Second, third, and fourth time-point samples from the same subjects were discarded. The resulting dataset of read counts containing 2,105 samples is reported in Additional file 12, which represents 210 ± 7 samples per body site. Further analysis of the dataset was performed using the per sample normalization to relative abundances. In the text, mean values are presented with standard deviation. The number of subjects with samples in the digestive tract retained for the 16S rRNA-based analysis was 209 post-quality control, from which 147 had sample data for all 10 body sites post-quality control. Unless otherwise noted, only first visit samples were used in all analyses.

### Biomarker discovery and visualization

The characterization of functional and organismal features differentiating the microbial communities specific to different body sites in the digestive tract was performed using our method for biomarker discovery and explanation called LEfSe [30]. LEfSe, publicly available [103], couples a standard test for statistical significance with a quantitative test for biological consistency, finally ranking the results by effect size. Briefly, it first uses the non-parametric factorial Kruskal-Wallis test to detect features (taxonomic clades or metabolic pathways) with abundances that differ below a significance threshold among groups of samples. Biological consistency is subsequently tested using the unpaired Wilcoxon rank-sum test among all pairs of sample groups; in our case this occurred between single body habitats. Finally, linear discriminant analysis (LDA) with bootstrapping is then used to rank differentially abundant features based on their effect sizes. A significance alpha of 0.05 and an effect size threshold of 2 were used for all biomarkers discussed in this study. Organismal and functional biomarkers are graphically represented here on hierarchical trees reflecting the RDP taxonomy [29] for 16S rRNA gene data and the KEGG BRITE hierarchy [49] on KEGG modules for metagenomic functional data.

### Clustering and statistical significance of four groups of body site habitats

For assessing bacterial community structure similarities between different samples and body sites, we compared the relative abundances of every pair of samples in our dataset using the Bray-Curtis measure of beta diversity [31]. The comparisons have been summarized in terms of within- and between-group averages as reported in Table 1; moreover, statistical significance has been tested for within versus between group distances, providing strong support (all *P*-values <10^-20^) for the clustering of all four groups in distinct community structures. A multidimensional scaling analysis was then performed on the Bray-Curtis diversity matrix and the four groups were denoted with different colors for highlighting the clustering structure (Additional file 4).

### Whole genome shotgun sequencing, read processing, and community metabolic profiling

Whole genome shotgun sequencing employed the Illumina GAIIx platform (Illumina, Inc.) as previously described [10]. The number of samples and nucleotide content from 98 subjects is summarized in Additional file 12. The abundances and presence (or absence) of pathways in these metagenomic data were inferred using the HUMAnN pipeline (HMP Unified Metabolic Analysis Network) [48]. Briefly, the metabolic and biomolecular potential of each sample was profiled starting from the 100 bp Illumina sequences after quality and length filtering. Reads were mapped to KEGG v54 orthologous gene families (KEGG KOs [49]) using MBLASTX (MulticoreWare, St. Louis, MO, USA), an accelerated translated BLAST implementation, using default parameters and a maximum E-value of 1. Hits were mapped to abundances of each KO using up to the 20 most significant hits, weighted by the quality of each hit (inverse blastx *P*-value) and normalized by the length of the hit gene. Pathway information was then recovered by assigning KO gene families to KEGG modules (representing small pathways of approximately 5 to 20 genes) using a combination of MinPath [104], filtering of pathways not consistent with the BLAST-derived taxonomic composition of the community, and gap filling of likely missing enzymes. The resulting KO and KEGG module relative abundances were used in the presented analysis. Further details of the HUMAnN methodology, its software implementation, and an extensive validation of each computational step appear in [48].

### Data accessibility

The datasets used in these analyses were deposited by the NIH Common Fund Human Microbiome Consortium at the Data Analysis and Coordination Center (DACC) for the Human Microbiome Project. Specifically, the downloadable packaged datasets are the 16S rRNA gene dataset [105], phylotype-classification of the 16S rRNA gene dataset [106,107], Human Microbiome Illumina whole genome shotgun reads [108], and the metabolic reconstruction tables [109]. The phylotype classification processed for normalization and quality control is available in Additional file 7.

## Abbreviations

HMP: Human Microbiome Project; HUMAnN: HMP Unified Metabolic Analysis Network; KEGG: Kyoto Encyclopedia of Genes and Genomes; KO: KEGG Orthology; LEfSe: LDA Effect Size; RDP: Ribosome Database Project.

## Competing interests

The authors declare that they have no competing interests. While the National Institutes of Health were one of the major drivers for the creation of the Human Microbiome Project, the NIH had no role in data analysis, decision to publish, or preparation of the manuscript.

## Authors' contributions

CH, DG, JI, KPL, LW, NS, PM, and SKH analyzed the data. CH, DG, NS, and JI contributed analysis tools. CH, JI, KPL, PM and SKH wrote the paper. All authors have read and approved the manuscript for publication.

## Supplementary Material

Additional file 1 **Table s1 - average abundance, expressed in percentage of all microbial clades in the four digestive tract groups and among the ten body habitats**. Lettering of groups and body habitats are as in Figure 1. AVG, average; STDEV, standard deviation. Click here for file

Additional file 2 **Table s2 - **s**urfaces associated with the sampling sites from which the microbiota of the digestive tract was collected**. Click here for file

Additional file 3 **Figure s1 - higher resolution version of **Figure 1b**showing significantly enriched taxa from the four groups of digestive tract sites**. This circular cladogram reports significant group-enriched taxa. Differential taxa analysis was performed using LEfSe on all the samples. Colored shading highlights which of the four major bacterial phyla was most enriched in which of the four body site groups. Each colored dot indicates a taxon that was differentially abundant among the groups. Small letters denote bacterial families that were enriched in one of the four body site groups. Click here for file

Additional file 4 **Figure s2 - diversity-based multidimensional scaling (MDS) plot of samples**. A distance matrix for all pairwise distances between samples was calculated using Bray-Curtis distance, which was used to project samples to MDS coordinates using the stats::cmdscale R function with default options. Each of the four established groups of body sites (G1, G2, G3, G4) is assigned a color, decreasing in opacity as the density of points of that group decreases, and body sites are denoted with different marker types. G2 and G3 contain the most overlap, while maintaining distinct areas of highest density, while G1 and G4, respectively, increase in distinctness. The distribution of samples in specific body sites does not produce sub-clusters, confirming the homogeneity of bacterial community composition within the four groups. Click here for file

Additional file 5 **Table s3 - inverse Simpson for each habitat of the digestive tract**. The minimum, maximum, average and standard deviation values are reported. Click here for file

Additional file 6 **Table s4 - percentages of subjects for whom each taxon was detected in both the upper digestive tract and in the stool**. The table is ordered based on the absolute differences between the presence in the stool and in at least one oral body site. Only the subjects with samples in all ten digestive tract body habitats were considered (n = 147) and all the taxonomic units with at least 40% of presence in stool or any oral body site are included. Click here for file

Additional file 7 **Table s5 - read counts for all digestive tract samples (after quality control) for each microbial clade**. Click here for file

Additional file 8 **Figure s3 - visual and schematic representation of the oral cavity and oropharyngeal sampling sites**. The soft tissues, illustrated here in a 20-year-old healthy male, were sampled by swabbing the tongue dorsum, hard palate, right and left buccal mucosa, the anterior keratinized gingiva, the right and left palatine tonsils, and the throat (posterior wall of the oropharynx). The pooled supragingival and pooled subgingival plaque samples were taken with curettes from molars, premolars and incisors (schematic illlustration). Not shown is the sampling of the saliva, which was collected by having the subject drool accumulated saliva into a collection vial. The complete sampling procedure is described in the Manual of Procedures for Human Microbiome Project (see Materials and methods). Click here for file

Additional file 9 **Figure s4 - higher resolution version of **Figure 6**showing functional characterization of the digestive microbiota**. Differentially abundant metabolic pathways from the buccal mucosa, supragingival plaque, tongue dorsum, and stool are depicted based on metabolic profiling performed with HUMAnN [48] from metagenomic shotgun sequencing data. Lettering indicates metabolic modules. Nucleot./amino acid met., nucleotide and amino acid metabolism; Carbohydrate/lipid met., carbohydrate and lipid metabolism; Energy met., energy metabolism; Met., aminoacyl tRNA and nucleotide sugar metabolism; Genetic information proc., genetic information processes; Environmental inf. proc., environmental information processing. Click here for file

Additional file 10 **Table s6 - percentages of metagenomic reads assigned to Archaea, Bacteria, and non-human Eukaryota (human reads excluded) in the four digestive tract sites with more than 50 shotgun sequencing samples available**. Click here for file

Additional file 11 **Figure s5 - a subset of significant correlations between metagenomic gene family and organismal abundances**. Paired shotgun metagenomic and 16S rRNA gene sequencing samples were associated, resulting in 34 buccal mucosa, 35 stool, 33 supragingival plaque, and 30 tongue microbiomes for joint analysis. Within each body site, Spearman correlations were calculated between the 21 KEGG Orthology gene families described in the Results and all phylotypes at any taxonomic level from phylum to OTU. Significant associations reaching a Benjamini-Hochberg false discovery rate <0.05 are shown here; grey ellipses represent clades, white rectangles KO gene families, and edge width is proportional to -log(q-value). Colors are as in Figure 1 (red, buccal mucosa; green, stool; yellow, plaque; blue, tongue). Click here for file

Additional file 12 **Table s7 - summary of the read statistics for 16S rRNA gene taxonomic abundances and whole genome shotgun sequencing metagenomic data**. Click here for file
