# Supplementary figures and images for: Composition of the adult digestive tract bacterial microbiome based on seven mouth surfaces, tonsils, throat and stool samples
Source: Genome Biol. 2012 Jun 14;13(6):R42. doi: 10.1186/gb-2012-13-6-r42 (PMC3446314; doi:10.1186/gb-2012-13-6-r42)

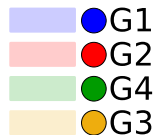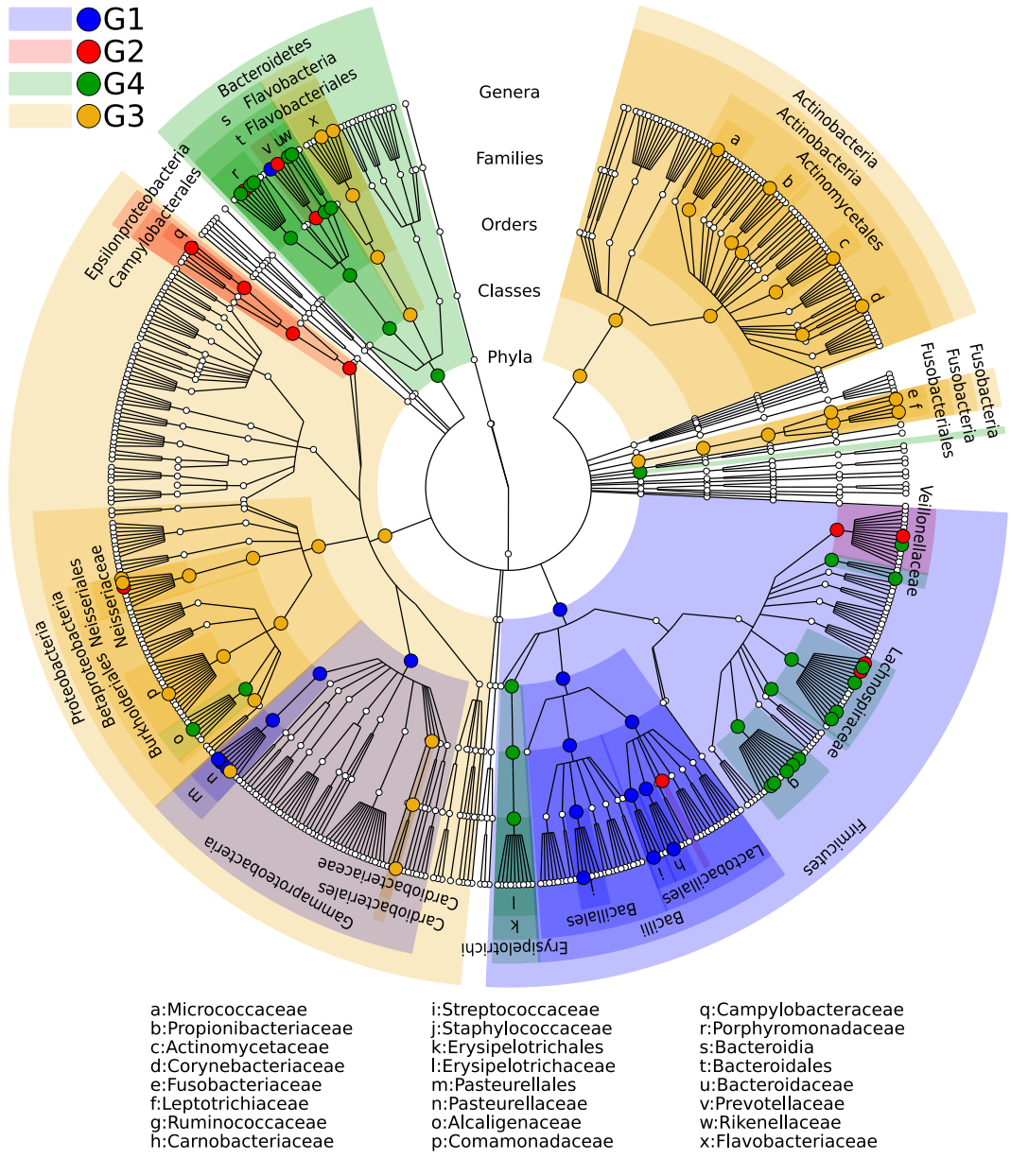

Supplement: Additional file 3 — Figure s1 - higher resolution version of Figure 1bshowing significantly enriched taxa from the four groups of digestive tract sites. This circular cladogram reports significant group-enriched taxa. Differential taxa analysis was performed using LEfSe on all the samples. Colored shading highlights which of the four major bacterial phyla was most enriched in which of the four body site groups. Each colored dot indicates a taxon that was differentially abundant among the groups. Small letters denote bacterial families that were enriched in one of the four body site groups. [file gb-2012-13-6-r42-S3.PDF]

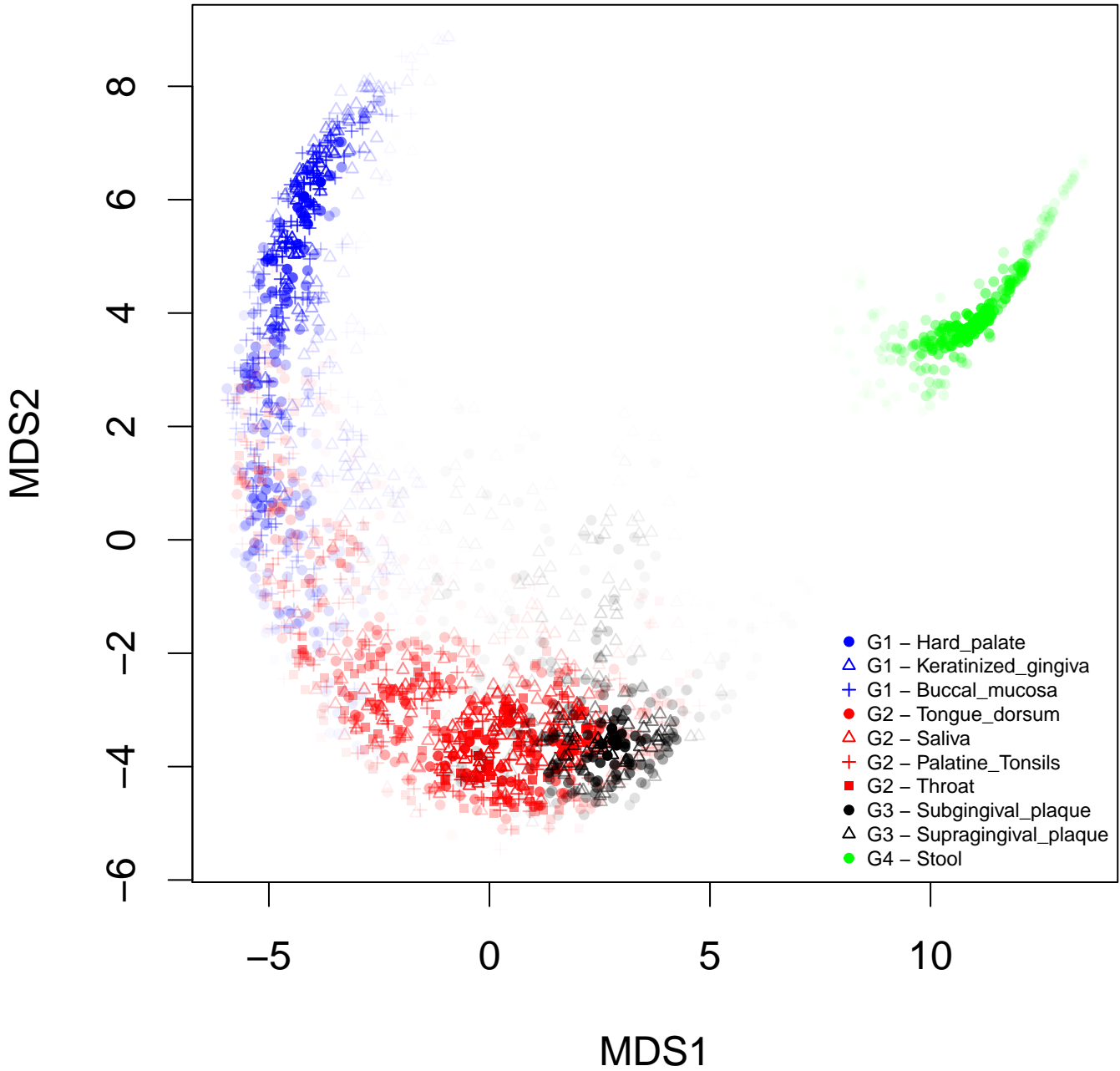

Supplement: Additional file 4 — Figure s2 - diversity-based multidimensional scaling (MDS) plot of samples. A distance matrix for all pairwise distances between samples was calculated using Bray-Curtis distance, which was used to project samples to MDS coordinates using the stats::cmdscale R function with default options. Each of the four established groups of body sites (G1, G2, G3, G4) is assigned a color, decreasing in opacity as the density of points of that group decreases, and body sites are denoted with different marker types. G2 and G3 contain the most overlap, while maintaining distinct areas of highest density, while G1 and G4, respectively, increase in distinctness. The distribution of samples in specific body sites does not produce sub-clusters, confirming the homogeneity of bacterial community composition within the four groups. [file gb-2012-13-6-r42-S4.PDF]

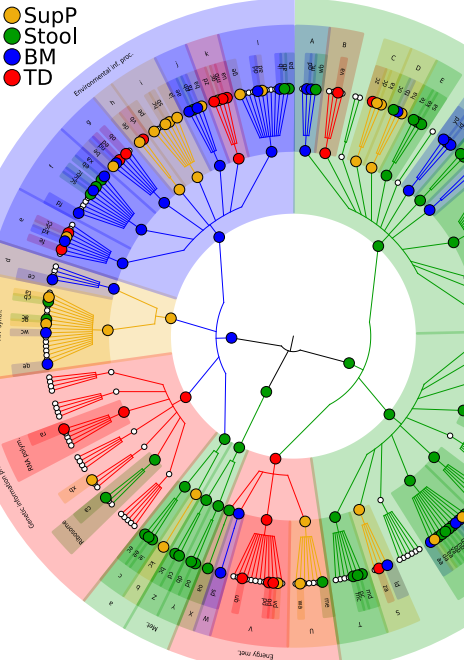[illegible][illegible][illegible]

Supplement: Additional file 9 — Figure s4 - higher resolution version of Figure 6showing functional characterization of the digestive microbiota. Differentially abundant metabolic pathways from the buccal mucosa, supragingival plaque, tongue dorsum, and stool are depicted based on metabolic profiling performed with HUMAnN [48] from metagenomic shotgun sequencing data. Lettering indicates metabolic modules. Nucleot./amino acid met., nucleotide and amino acid metabolism; Carbohydrate/lipid met., carbohydrate and lipid metabolism; Energy met., energy metabolism; Met., aminoacyl tRNA and nucleotide sugar metabolism; Genetic information proc., genetic information processes; Environmental inf. proc., environmental information processing. [file gb-2012-13-6-r42-S9.PDF]

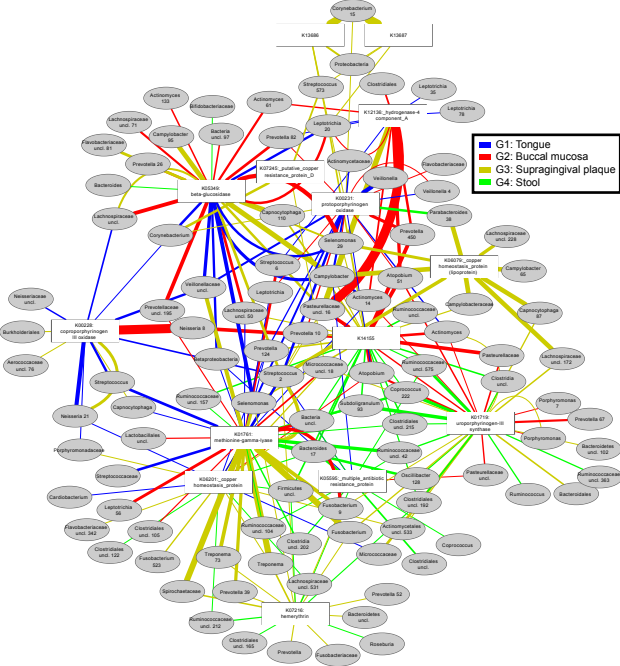

Supplement: Additional file 11 — Figure s5 - a subset of significant correlations between metagenomic gene family and organismal abundances. Paired shotgun metagenomic and 16S rRNA gene sequencing samples were associated, resulting in 34 buccal mucosa, 35 stool, 33 supragingival plaque, and 30 tongue microbiomes for joint analysis. Within each body site, Spearman correlations were calculated between the 21 KEGG Orthology gene families described in the Results and all phylotypes at any taxonomic level from phylum to OTU. Significant associations reaching a Benjamini-Hochberg false discovery rate <0.05 are shown here; grey ellipses represent clades, white rectangles KO gene families, and edge width is proportional to -log(q-value). Colors are as in Figure 1 (red, buccal mucosa; green, stool; yellow, plaque; blue, tongue). [file gb-2012-13-6-r42-S11.PDF]
