# Supplementary material for: Composition of the adult digestive tract bacterial microbiome based on seven mouth surfaces, tonsils, throat and stool samples
Source: Genome Biol. 2012 Jun 14;13(6):R42. doi: 10.1186/gb-2012-13-6-r42 (PMC3446314; doi:10.1186/gb-2012-13-6-r42)

# Oral Cavity Sampling Sites

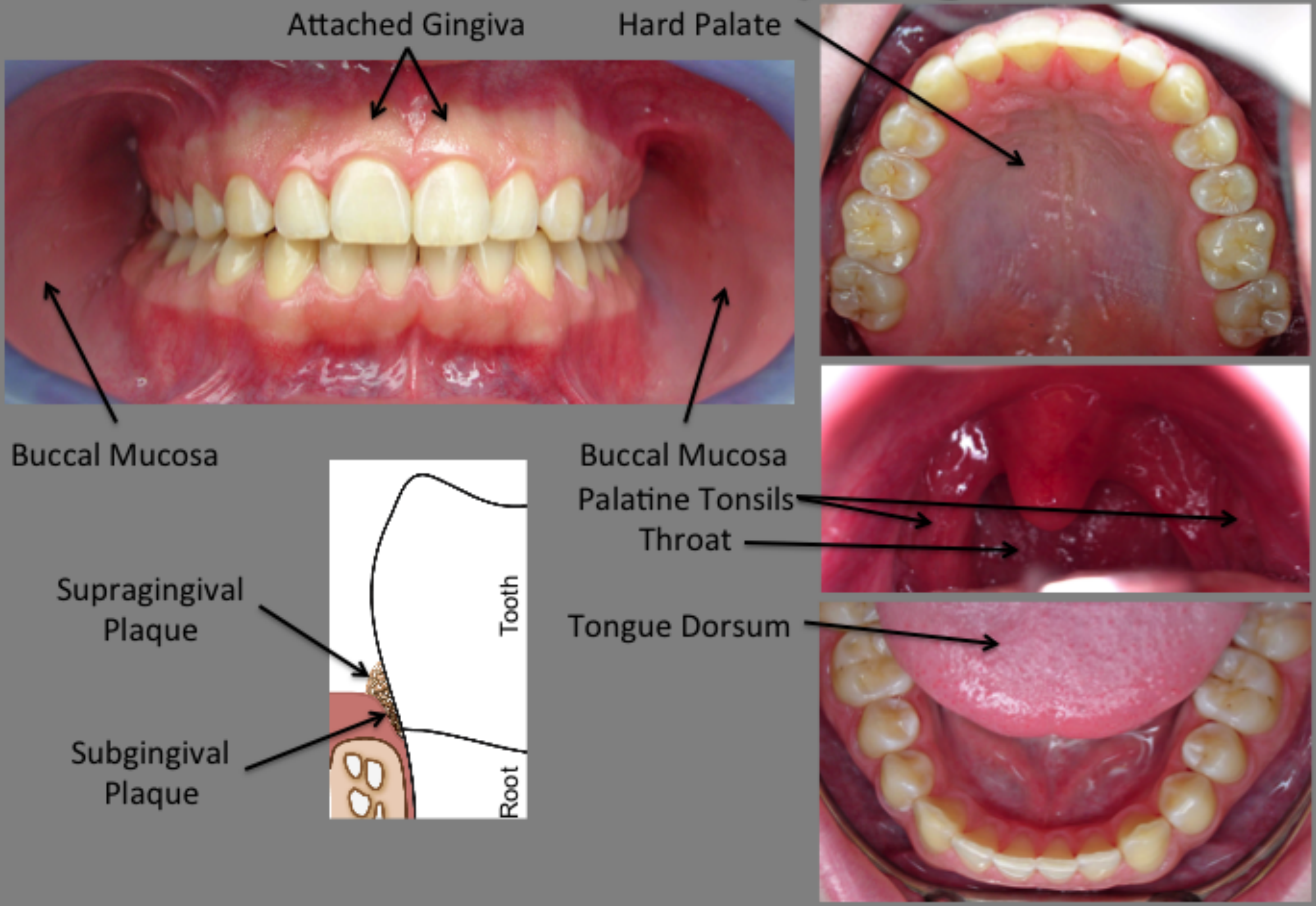

Supplement: Additional file 8 — Figure s3 - visual and schematic representation of the oral cavity and oropharyngeal sampling sites. The soft tissues, illustrated here in a 20-year-old healthy male, were sampled by swabbing the tongue dorsum, hard palate, right and left buccal mucosa, the anterior keratinized gingiva, the right and left palatine tonsils, and the throat (posterior wall of the oropharynx). The pooled supragingival and pooled subgingival plaque samples were taken with curettes from molars, premolars and incisors (schematic illlustration). Not shown is the sampling of the saliva, which was collected by having the subject drool accumulated saliva into a collection vial. The complete sampling procedure is described in the Manual of Procedures for Human Microbiome Project (see Materials and methods). [file gb-2012-13-6-r42-S8.PDF]
